# Flavonoid Compounds and Photosynthesis in *Passiflora* Plant Leaves under Varying Light Intensities

**DOI:** 10.3390/plants9050633

**Published:** 2020-05-15

**Authors:** Yu-Wan Ni, Kuan-Hung Lin, Kai-Hsien Chen, Chun-Wei Wu, Yu-Sen Chang

**Affiliations:** 1 Department of Horticulture and Landscape Architecture, National Taiwan University, Taipei 106, Taiwan; choocle@yahoo.com.tw (Y.-W.N.); kaihchen@ntu.edu.tw (K.-H.C.); 2 Department of Horticulture and Biotechnology, Chinese Culture University, Taipei 111, Taiwan; rlin@faculty.pccu.edu.tw

**Keywords:** flavonoids, isovitexin, light intensity, orientin, passionfruit

## Abstract

Functional constituents in the leaves of *Passiflora* plants contain antidepressant and antianxiety effects which are beneficial to human health and fitness. The objective of this study was to investigate leaf growth, physiological parameters, and secondary metabolite contents of Tainung No. 1 variety (*P. edulis × P. edulis f. flavicarpa*.) and *P. suberosa* in response to three light intensity conditions, including 100% light intensity (LI-100), 50% light intensity (LI-50), and 15% light intensity (LI-15) for 2 months. The leaf number, length, width, area, dry weight (DW), minimal fluorescence (Fo), maximal fluorescence (Fm), maximum photochemical efficiency of photosystem II, and soil-plant analysis development (SPAD) values of all tested plants increased with a decreasing light intensity, except for the leaf number and DW of *P. suberosa* plants. Low values of the net photosynthetic rate, transpiration rate, and stomatal conductance of Tainung No. 1 leaves in the LI-15 treatment showed the acclimation capacity of these plants. These observations together with high values of leaf growth traits of Fo, Fm, SPAD, and the intercellular-to-atmospheric CO_2_ concentration ratio indicate their physiological plasticity, which is of fundamental importance when cultivating plants in environments with different light availabilities. Wide variations occurred in total phenol (TP), total flavonoid (TF), orientin (OR), and isovitexin (IV) contents of the two *Passiflora* varieties, and *P. suberosa* contained higher TP and TF contents than did Tainung No. 1 in each light treatment but IV content of *P. suberosa* was lower than that of Tainung No. 1 in the LI-15 treatment. Moreover, increases in TF, OR, and IV contents of Tainung No. 1 and *P. suberosa* were clear in the LI-50 and LI-100 treatments, respectively, compared to LI-15 treatment. Leaf growth, physiological parameters, and secondary metabolite accumulations in *Passiflora* species can be optimized for commercial production via lighting control technologies, and this approach may also be applicable to leafy vegetables to produce a stable industrial supply of high leaf yields and metabolite contents.

## 1. Introduction

The genus *Passiflora* (family Passifloraceae) includes more than 400 plant species distributed in tropical and subtropical regions of the world [1]. Passionfruit species are commercially important in the juice industry and are used for nutritional consumption. In addition, because their pharmacological properties are known to popular medicine, passionfruit varieties are considered functional foods. Extracts of leaves of *Passiflora* species are used in folk medicine to treat diabetes, hypertension, skin diseases, anxiety, irritability, migraines, insomnia, opiate withdrawal, and attention deficit hyperactivity [2]. Ethnobotanical studies showed that the fruit pulp of *Passiflora* species is used as a cardiac tonic, a moderate diuretic, and digestive stimulant and to treat asthma, bronchitis, whooping cough, and urinary infections [3].

Light is an important environmental signal and induces chlorophyll (Chl) biosynthesis [4]. Chlorophyll fluorescence (ChlF) is a noninvasive technique and offers highly accurate measurements that illustrate the functioning of the photosynthetic apparatus in plants. Changes in light irradiance evoke variable morphogenetic and photosynthetic responses that vary among different plant species. Such photo-responses are of practical importance in modern plant cultivation technologies, since the feasibility of purposefully tailoring light intensities enables one to control plant growth, development, and nutritional quality [5]. The study of photosynthesis irradiance relationships is a basic aspect of plant physiological research and is important in managing various species, and photosynthetic light responses can be used to assess the ability to capture light and to understand the optimal light-intensity conditions of the habitats of plants [6]. Various light-intensity levels influence the photosynthetic efficiency of ornamental *Passiflora* flower hybrids [4]. Many photosynthetic parameters are related to the adaptability of plants to shade, including increases in Chl contents, the net photosynthetic rate (P_N_), the maximal quantum yield of photosystem II (PSII) photochemistry (Fv/Fm), and the intercellular-to-atmospheric CO_2_ concentration ratio (Ci/Ca) and decreases in stomatal conductance (Gs) and the transpiration rate (E) [7,8,9]. These changes maximize light interception and increase carbon gains at low light intensities, through more efficient investment in the photosynthetic machinery [10,11]. However, photosynthetic studies of *Passiflora* species have not been conducted, and understanding the adaptability mechanisms of these plants to various light intensities would be of great importance to the medicinal marketing of these plants.

Phenolic compounds, particularly flavonoids, are an important group of plant secondary metabolites, and the accumulation of different types of flavonoids, such as flavonols, flavones, and anthocyanins, helps plants cope with a wide variety of biotic and abiotic stresses [12,13]. As an important ecological factor, light affects the photosynthesis of plants and has a bearing on their chemical compositions [14]. Flavonoid biosynthesis is light dependent, and higher light intensities stimulate the synthesis of phenols and flavonoids to protect living plants [15]. Greater exposure to sunlight increases flavonoid contents and influences the color and nutritional quality of the fruit [16]. Moreover, Xu et al. [17] also reported that full sunlight can promote flavonol biosynthesis in leaves of *Gingko biloba*. Flavonoid biosynthesis and accumulation in plants are related to genetics and to environmental factors, and changes in light intensity can induce the synthesis and accumulation of secondary metabolites in soybeans [18], *Camptotheca acuminate* [19], *Epimedium pseudowushanense* [20], *Lithocarpus litseifolius* [21], *Stevia rebaudiana* [22], buckwheat [23], berries [24], *Ilex paraguariensis* [25], *Mikania micrantha*, *Tridax procumbens* [26], sweet basil [27], *Moringa oleifera* [28], *Syringa oblate* [29], and edible fern species [30]. However, the accumulation of total phenol (TP) contents and total flavonol (TF) compounds of *Passiflora* species grown under various light intensities has not yet been studied. Flavonols in plant tissues are mostly in the form of *O*-glycosides. Flavones are not widely distributed and are mainly represented in the plants by *C*-glucosides of apigenin and luteolin [31]. The major compounds present in *Passiflora* species are C-glycosyl flavonoids, such as isovitexin (IV, apigenin-6-*C*-glucoside) and orientin (OR, luteolin-8-*C*-glucoside) [32]. The pharmacological effects of IV and OR were previously described [33,34]. It is unknown whether concentrations of IV and OR in *Passiflora* species are affected by different light intensities. Thus, the contents of these particular flavonoids in the leaves of *Passiflora* species were examined in this study.

Several secondary metabolites were reported to occur in *P. edulis* [35]. *Passiflora suberosa* L. is native to the Americas and was introduced to Asia and Oceania, including Taiwan, but phenol and flavonoid concentrations of this species have never been studied. The observed changes in phenol and flavonoid concentrations were compared between the two species evaluated. The hypothesis of this research was that some photosynthetic components and the production of secondary metabolites in leaves might exhibit distinguishable differences between plant species under different light intensities. The aims of this study were to investigate effects of light intensities on leaf growth, photosynthetic parameters, and secondary metabolite accumulation in these *Passiflora* species. Application of photosynthetic indices can efficiently determine light intensity-induced changes in phenol and flavonoid contents in *Passiflora* species and allows the exploration of specific varietal responses to lighting stress. This research can also provide a scientific basis for the cultivation and management of these *Passiflora* species and can contribute to our understanding of acclimation by *Passiflora* plants to optimal levels of light irradiance for growing these plants.

## 2. Results and Discussion

### 2.1. Light Intensity Effects on Leaf Growth Traits of Passiflora Species

**Figure 1:**
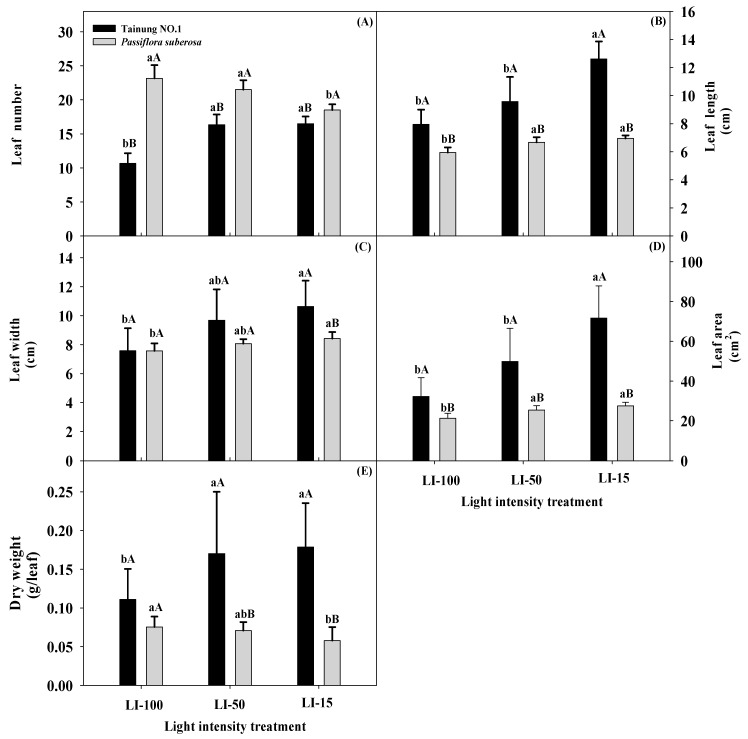
Effects of light intensity on the leaf number (**A**), length (**B**), width (**C**), area (**D**), and dry weight (**E**) of Tainung No. 1 and *Passiflora suberosa* plants. Data were recorded and calculated after 2 months of light-intensity treatment of six replicates. Means within a light treatment followed by different lowercase letters significantly differ at *p* ≤ 0.05 by the least significant difference (LSD) test. Means within the same light treatment of the two species followed by different capital letters significantly differ at *p* ≤ 0.05 by the LSD test. Each treatment was assumed to be dependent on the other. LI-100 (100% light intensity, non-shaded, as the control), LI-50 (50% light intensity, 50% shaded), and LI-15 (15% light intensity, 85% shaded) represent the average light intensity passing through in each treatment at 1396, 619, and 187 μmol m^−^^2^s^−^^1^ photosynthetic photon flux density (PPFD), respectively.

Table 1 illustrates how leaf growth and DW of plants differed in the two species of *Passiflora* species after 2 months of cultivation at three different light-intensity treatments. All leaf growth parameters displayed significant differences (*p* ≤ 0.001 and 0.01) for the main effects (both V and L) and their interaction effects (L × V), with the exception of DW in the variety effect and leaf width and DW in interaction effects which showed nonsignificant differences. Significantly lower leaf numbers were detected in Tainung No. 1 (a variety of *P. edulis × P. edulis f. flavicarpa*) plants (10.67) and *P. suberosa* plants (18.50) under LI-100 and LI-15 treatments, compared to other lighting treatments (Figure 1 and Table S1). In addition, leaf numbers of Tainung No. 1 plants were significantly lower than those of *P. suberosa* plants under all light treatments, indicating that leaf numbers were affected by both light irradiance and genotype. In Tainung No. 1, significantly longer (12.61 cm) and wider (10.63 cm) leaves and LA (71.63 cm^2^) were detected with LI-15 compared to LI-100 treatment (Figure S1). Similar trends of leaf length and width and LA were also observed in *P. suberosa* plants, and maximal and significant increases in leaf length, width, and area (LA) occurred in the LI-15 treatment at 6.95 cm, 8.42 cm, and 27.50 cm^2^, respectively, compared to LI-100 treatment. When genotypes were compared across light treatments, Tainung No. 1 plants exhibited significantly longer leaves (7.94–12.61 cm) and larger LAs (32.28–71.63 cm^2^) than *P. suberosa* plants (5.95–6.95 cm and 21.21–27.50 cm^2^, respectively) in all light treatments, but no significant difference in leaf widths was observed in either genotype in any light treatments, except that significantly wider leaves occurred with LI-15 treatment in Tainung No. 1 plants (10.63 cm) compared to *P. suberosa* plants (8.42 cm). Tainung No. 1 had significantly lower DW (0.11 g leaf^−1^) in LI-100 compared to other light treatments (0.17 g leaf^−1^), whereas DW with LI-15 treatment (0.06 g leaf^−1^) was significantly less than that with LI-100 treatment (0.08 g leaf^−1^) in *P. suberosa* plants. Moreover, Tainung No. 1 plants had significantly higher leaf DWs in both LI-50 and LI-15 treatments compared to *P. suberosa* plants.

We detected significantly higher leaf number, length, width, and area in both species subjected to LI-15 irradiance compared to LI-100 irradiance, except *P. suberosa* plants had the maximal leaf number under LI-100 irradiance. All leaf growth traits (leaf number, length, width, area, and DW) increased in all plants as the light intensity decreased, except the leaf number and DW of *P. suberosa* plants decreased as the light intensity decreased, indicating that a low light intensity induced an increase in leaf growth traits of Tainung No. 1 plants. The increase in LA was caused by changes in leaf length and width in response to an increasing shaded level. Under low irradiance, Tainung No. 1 plants compensated for the decrease in light, making better use of this resource by increasing LA and efficient utilization of photoassimilates, as a larger photosynthetic area is produced per unit of accumulated DW [36]. Many types of morphologic and physiological stresses occur when plants encounter a light irradiance stress, and the tested plants’ visual appearances showed obvious changes after 8 weeks of low-light-intensity stress treatments. Visual observations indicated that the optimal growth and development of Tainung No. 1 and *P. suberosa* produced plants with acceptable leaves at LI-50 (50% shading) and LI-100 (no shading, control), respectively. Most upper and middle portions of leaves in Tainung No. 1 plants appeared healthy and green when they were grown under both LI-50 and LI-15 (85% shading) conditions compared to the LI-100 condition for 2 months, so those plants tended to be unaffected and exhibited adaptive morphologic plasticity. On the contrary, the growth of *P. suberosa* plants tended to be more sensitive to LI-15 than to the control, showing decreased leaf numbers and DW and visually higher light irradiance in the upper and middle portions of leaves of those plants under LI-15 compared to the control.

### 2.2. Effects of Different Light Intensities on Physiological Parameters in Passiflora Species

**Figure 2:**
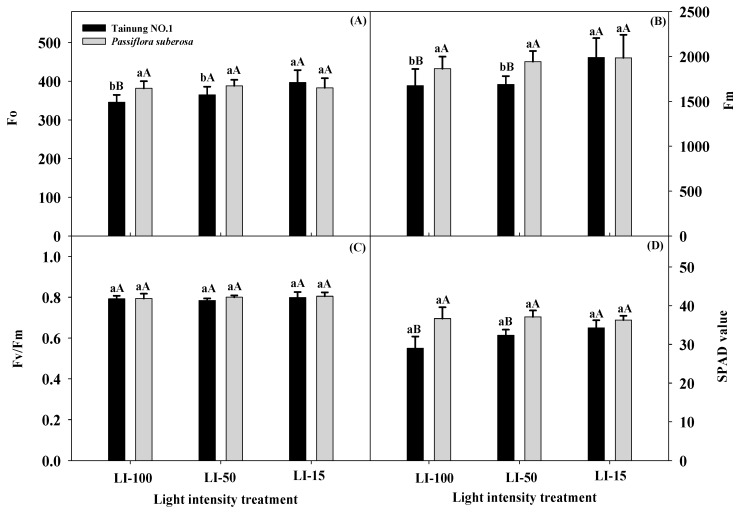
Effects of light intensity on minimal fluorescence (Fo) (**A**), maximal fluorescence (Fm) (**B**), maximum photochemical efficiency of photosystem II (Fv/Fm) (**C**), and soil-plant analysis development (SPAD) values (**D**) of Tainung No. 1 and *Passiflora suberosa* plants. Data were recorded and calculated after 2 months of light-intensity treatment of six replicates. Means within a light treatment followed by different lowercase letters significantly differ at *p* ≤ 0.05 by the least significant difference (LSD) test. Means within the same light treatment of the two species followed by different capital letters significantly differ at *p* ≤ 0.05 by the LSD test. Each treatment was assumed to be dependent on the other. LI-100 (100% light intensity, non-shaded, as the control), LI-50 (50% light intensity, 50% shaded), and LI-15 (15% light intensity, 85% shaded) represent the average light intensity passing through in each treatment at 1396, 619, and 187 μmol m^−^^2^s^−^^1^ PPFD, respectively.

Figure 2 shows the differential responses of both varieties towards different ChlF and total Chl (SPAD) values for different light treatments. ANOVA results of the main effects of light intensity treatment (L), variety (V), and their interaction effect (L × V) on fluorescence parameters and SPAD values are summarized in Table 1. All of the photosynthetic capacity and fluorescence parameters significantly differed at the levels of 0.1% or 5% for the main effects, except for Fo and Fv/Fm of the variety treatment and Fv/Fm of the light treatment, which showed negligible differences. Additionally, Fm and Fv/Fm indices did not significant differ in any light treatments with the different varieties. All ChlF parameters (Fo, Fm, and Fv/Fm) and SPAD values increased in all plants as the light intensity decreased, suggesting that long-term low-light-intensity treatment increased these values in both species. Both Fo and Fm values of Tainung No. 1 plants under LI-100 and LI-50 treatments were significantly lower (345.83–364.07 and 1674.17–1687.67, respectively) than those of LI-15 treatments (396.67 and 1989.33, respectively), but no significant differences in either Fo or Fm values of *P. suberosa* plants were observed in any light treatment (Table S2). In addition, it is noteworthy that, when different light-intensity treatments across varieties were compared, Tainung No. 1 exhibited significantly lower Fo and Fm values (345.83 and 1674.17, respectively) than *P. suberosa* under LI-100 treatment (381.67 and 1863.83, respectively). Fv/Fm values of all plants with all light treatments showed no significant differences, suggesting that Fv/Fm is not a sufficient parameter to determine adaptability of these tested plants to light intensities. SPAD values of all tested plants displayed no significant differences in any lighting treatment, indicating that this parameter was not affected by different light conditions. Nevertheless, SPAD levels of *P. suberosa* plants grown under LI-100 and LI-50 conditions (36.66 and 37.10, respectively) were significantly higher than those in Tainung No. 1 plants (29.00 and 32.35, respectively).

**Figure 3:**
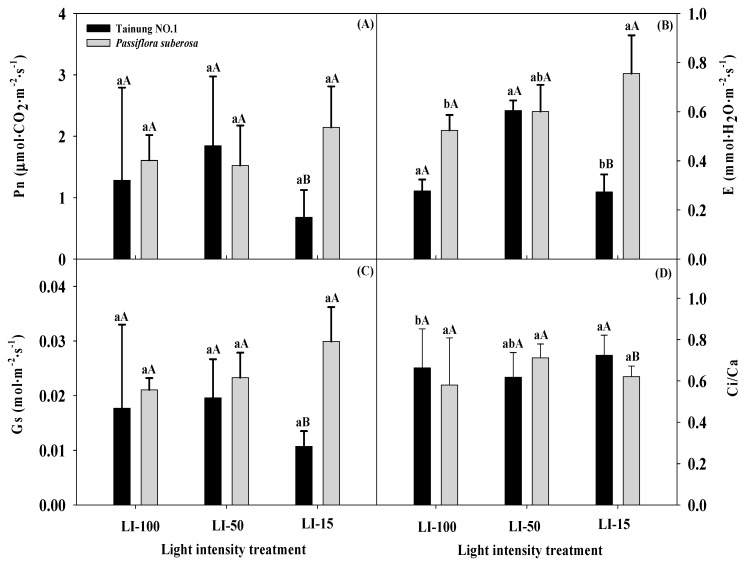
Effects of light intensity on the net photosynthetic rate (Pn) (**A**), transpiration rate (E) (**B**), stomatal conductance to water vapor (Gs) (**C**), and intercellular-to-atmospheric CO_2_ concentration ratio (Ci/Ca) (**D**) of Tainung No. 1 and *Passiflora suberosa* plants. Data were recorded and calculated after 2 months of light-intensity treatment of six replicates. Means within a light treatment followed by different lowercase letters significantly differ at *p* ≤ 0.05 by the least significant difference (LSD) test. Means within the same light treatment of the two species followed by different capital letters significantly differ at *p* ≤ 0.05 by the LSD test. Each treatment was assumed to be dependent on the other. LI-100 (100% light intensity, non-shaded, as the control), LI-50 (50% light intensity, 50% shaded), and LI-15 (15% light intensity, 85% shaded) represent the average light intensity passing through in each treatment at 1396, 619, and 187 μmol m^−^^2^s^−^^1^ PPFD, respectively.

Figure 3 reveals that values of photosynthetic capacity indices in the *Passiflora* varieties responded differently to lighting conditions, and all of the photosynthetic capacity indices significantly differed at the 1% or 5% levels for the main effect of variety (V) only (Table 1). There were no marked differences in Pn values of any tested plants among the different lighting treatments, except that a significantly higher Pn value of *P. suberosa* plants (2.14 μmol·CO_2_·m^−2^·s^−1^) was detected in the LI-15 treatment compared to that of Tainung No. 1 plants (0.68 μmol·CO_2_·m^−2^·s^−1^) (Table S3). Maximal E values of Tainung No. 1 and *P. suberosa* plants were detected with LI-50 (0.5 mmol·H_2_O·m^−2^·s^−1^) and LI-15 (0.76 mmol·H_2_O·m^−2^·s^−1^), respectively, and Tainung No. 1 plants grown under LI-15 irradiance had a significantly lower E value (0.27 mmol·H_2_O·m^−2^·s^−1^) than that of *P. suberosa* plants. No significant differences in Gs values of any tested plants at the eighth week were detected under any lighting treatment, but Tainung No. 1 plants (0.01 mol·m^−2^·s^−1^) displayed a significantly lower Gs value than *P. suberosa* plants (0.03 mol·m^−2^·s^−1^) under LI-15 treatment. The Ci/Ca value of Tainung No. 1 plants under LI-15 treatment (0.77) was significantly higher than that under LI-100 treatment (0.65), and this value was also significantly higher than that of *P. suberosa* plants (0.62) when species were compared across light treatments.

SPAD values have been widely examined in a number of plants to determine injury or tolerance to various environmental stresses, including light radiation, and SPAD assesses total Chl contents and the photosynthetic capacity [37]. The integrated regulation of the entire photosynthesis process takes place in such a way that an internal balance between the efficiency of the photosynthetic light phase reaction and the efficiency of reactions leading to CO_2_ assimilation is maintained [38]. In addition, Mauro et al. [39] demonstrated that a decrease in the light intensity occurred at the expense of electron transport, photophosphorylation, and carbon fixation components, resulting in a reduced ability of leaves to assimilate CO_2_. Reed et al. [40] also illustrated that the entire photosynthetic apparatus in the shade is more efficient at harvesting light, but it assimilates less CO_2_ compared to leaves in the sun. Plants adapted to shaded environments can achieve a photosynthetic rate equal to half that measure in the sun, and characteristics such as increasing the efficiency of uptake and use of the available radiation and low rates of carbon losses through respiration reflect a survival strategy developed by plants adapted to environments with limited light intensities [41]. In this study, the lower Gs value (0.01 mol·m^−2^·s^−1^) of Tainung No. 1 plants under LI-15 treatment was limiting in the rate of CO_2_ fixation due to a consequent decrease of its concentration in stomatal cavities and intercellular spaces, and thus, a low Pn at 0.68 μmol·CO_2_·m^−2^·s^−1^ photosynthetic photon flux density (PPFD) was maintained. Conditions of low light intensity (15%) were favorable for minimizing Gs values of Tainung No. 1 plants, which also contributed to a reduction in the E value at 0.27 mmol·H_2_O·m^−2^·s^−1^ PPFD and a high value of the Ci/Ca ratio (0.77). Abreu et al. [4] reported that shade-tolerant *Passiflora* “Aninha” and *Passiflora* “Priscilla” passion fruit plants had higher Ci/Ca ratios under low light intensities. The Ci/Ca ratio is an indicator of stomatal limitation of photosynthesis, and this ratio changes according to the habitat, with shade-tolerant species presenting higher Ci/Ca values than those of sun-adapted species [36]. Tainung No. 1 plants survived at LI-15 and had higher leaf growth traits of Fo, Fm, SPAD, and Ci/Ca values and lower Pn, E, and Gs values, indicating that such species may persist in such conditions, but cultivation in full sunlight (LI-100) did not seem to be the most proper condition for the growth and development of these plants. These results suggest that physiological plasticity to different intensities of light, which helps maintain a positive carbon balance under conditions of low light intensities and shading treatment, could diminish the water transpiration rate and net photosynthesis while maintaining photoreduction and oxidation of plastoquinone acceptors associated with PSII inactivation in tylakoidal membranes [4], thereby allowing Tainung No. 1 plants to survive and function during 85% shading. Therefore, Tainung No. 1 plants were able to acclimatize to low irradiances, seemed to be a shade-tolerant species showing adaptability to shade, and could be used for indoor landscaping projects. Nevertheless, long-term LI-15 treatment in *P. suberosa* plants may cause reductions in CO_2_ assimilation and leaf growth and development retardation, in which both the leaf number and DW would significantly decrease with an increase in E values under LI-15 treatment compared to the control, indicating that the low light intensity and water relationships of *P. suberos* plants were affected during low-light treatment. Lower values of Gs consequently prevent the egress of H_2_O molecule from the leaves, but a higher Gs value was expected with LI-15 treatment to control water loss by transpiration and assimilation of CO_2_, thereby maintaining Pn at an optimum level [4]. The photosynthetic apparatus of Tainung No. 1 plants grown in LI-15 reflected the selection pressure to maximize the absorption of light, while minimizing respiratory costs associated with a high photosynthetic capacity, whereas *P. suberosa* plants increased the susceptibility to an event of light stress and caused photoinhibition under an LI-15 condition [36]. During LI-15 treatment, stomata might have been closed by an inhibition of water absorption through the roots, although there was sufficient water in the pots. Reducing the luxury transpiration rate by applying shading treatments provides an opportunity to relieve adverse effects of suboptimal light intensities on *Passiflora* plants and to improve their photosynthesis capacity when transpiration frequently exceeds water uptake.

There is limited information available regarding the ecophysiological development of *Passiflora* plants grown under light irradiance stresses. One of the objectives of this study was to employ nondestructive measurements to determine the leaf total Chl content, ChlF, and photosynthetic capacity values and to develop precise, integrated, and quantitative measurements of *Passiflora* species under various light irradiance conditions. The different ChlF and photosynthetic capacity parameters are highly sensitive indicators representing the physiological status of stressed plants and provide a quick means of identifying a plant’s physiological condition [42]. The shading treatment could promote Tainung No. 1 leaf growth, and SPAD, Fo, and Fm values can be used for the rapid monitoring and early detection of light irradiance in the early growth stage and screening of individual plants that exhibit tolerance to low light irradiance. These photosynthetic parameters were specific for light irradiance stress and were not solely expressed in response to an increasing excess of photon energy. Different parameters acted differently under various light-intensity treatments; for instance, shading treatments can be applied to plants when SPAD values reach 35. However, each parameter is not necessarily equally significant in protecting against light-irradiance extremes. The applications of combined tools allow exploration and explanation on specific varietal responses to light irradiance. Combining SPAD (>34), Fo (>396), and Fm (>1989) values after shading treatment is applicable to comprehensively measure leaf growth and to select against the most susceptible plants when developing indices for nondestructive Chl estimation of plant leaves and can indicate plants’ photosynthetic efficiency and gas exchange measurements. This means that many hundreds of individual plants can be screened per day, providing more opportunities to discover individuals that manifest leaf growth and development indicators and to exhibit greater photosynthesis and transpiration. However, no studies have been conducted on the effects of light-intensity treatments on leaf growth, photosynthesis, and transpiration values of *Passiflora* species. Therefore, our results can be applied to improve the light-irradiance stress tolerance of *Passiflora* plants, developing management practices for field cultivation, reducing light energy consumption, and enhancing cultivation when light resources are limited. In addition, a better understanding of the growth characteristics of these plants would aid their effective cultivation for farming in areas with extreme climates. Photosynthetically active radiation varies throughout habitats, inducing plants to adapt to those alterations. Within the plasticity possible in each environment, plants adjust their chlorophyll resources and photosynthetic traits in response to the light intensity. As such, plants in the sun and those in the shade differ in various aspects. Thus, shading treatments may activate photosynthesis and transpiration values in *Passiflora* species, which offers insights into their mechanisms for light-saving advances in the future.

### 2.3. Influences of Light Intensity Effects on Secondary Metabolites of Passiflora Species

**Figure 4:**
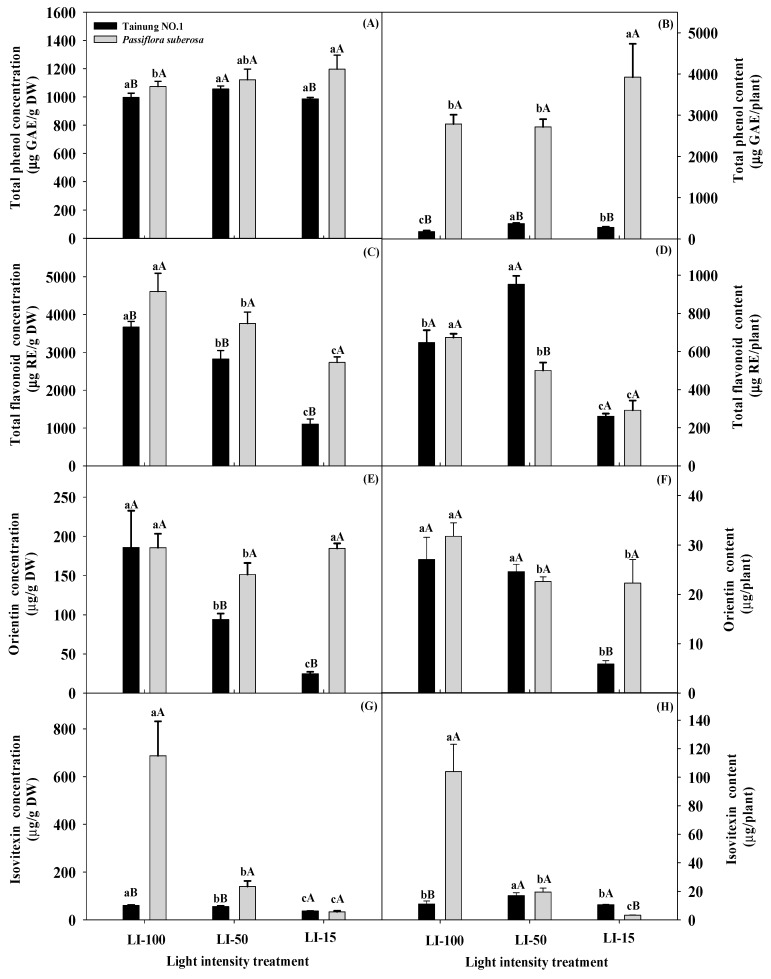
Effects of the light intensity on contents of total phenol (**A**,**B**), total flavonoid (**C**,**D**), orientin (**E**,**F**), and isovitexin (**G**,**H**) per leaf (left panels) and per plant (right panels) in Tainung No. 1. and *Passiflora suberosa* plants: Data were recorded and calculated after 2 months of light-intensity treatment of six replicates. Means within a light treatment followed by different lowercase letters significantly differ at *p* ≤ 0.05 by the least significant difference (LSD) test. Means within the same light treatment of the two species followed by different capital letters significantly differ at *p* ≤ 0.05 by the LSD test. Each treatment was assumed to be dependent on the other. LI-100 (100% light intensity, non-shaded, as the control), LI-50 (50% light intensity, 50% shaded), and LI-15 (15% light intensity, 85% shaded) represent the average light intensity passing through in each treatment at 1396, 619, and 187 μmol m^−^^2^s^−^^1^ PPFD, respectively.

In Table 2, all secondary metabolite contents displayed significant differences (*p* ≤ 0.001, 0.01, and 0.05) for the main and interaction effects, with the exception of the TP content (per DW) in the variety effect. Figure 4 presents levels of the TP, TF, OR, and IV in leaf extracts of different varieties under various light irradiances, showing that the trends and rates of increase in TP, TF, OR, and IV under various light intensities differed among the species. For example, no significant differences in leaf TP concentration were observed in Tainung No. 1 plants (1001.89–1048.48 μg GAE (g DW)^−1^) subjected to all lighting treatments, whereas *P. suberosa* plants subjected to LI-15 treatment (1196.62 μg GAE (g DW)^−1^) exhibited a remarkably higher TP concentration compared to LI-100 treatment (1073.27 μg GAE (g DW)^−1^) (Table S4). When species were compared across light treatments, TP levels of *P. suberosa* plants under LI-100 and LI-15 treatments (at 1073.27 and 1196.62 μg GAE (g DW)^−1^, respectively) were significantly higher than those of Tainung No. 1 plants (at 1001.89 and 1014.90 μg GAE (g DW)^−1^, respectively). Thus, different species displayed variations in their TP contents. When the TP content was calculated at the plant level, Tainung No. 1 plants had significantly higher TP contents in response to LI-50 treatment (364.85 μg GAE per plant) compared to other light treatments (187.90 and 294.76 μg GAE per plant), while in *P. suberosa* plants, a significantly higher TP content was observed in LI-15 treatment (3921.6 μg GAE per plant) compared to other treatments (2786.73 and 2717.29 μg GAE per plant). In addition, all *P. suberosa* plants had significantly higher TP contents than Tainung No. 1 plants in all light treatments, and in LI-100 treatment, the *P. suberosa* species (2786.73 μg GAE per plant) exhibited a 15-fold increase compared to the Tainung No. 1 species (187.90 μg GAE per plant).

TF concentration of both varieties significantly decreased as the light intensity decreased, and TP concentration of *P. suberosa* plants (2733.10–4611.73 μg RE (g DW)^−1^) were significantly higher than those of Tainung No. 1 plants (1072.97–3719.78 μg RE (g DW)^−1^) under all light treatments. When the TF content was calculated on the plant level, remarkably lower TP contents occurred under LI-15 treatment in both species (277.68 and 291.68 μg RE per plant, respectively) compared to other light treatments, and significantly higher TF contents were detected in Tainung No. 1 plants (934.07 μg RE per plant) compared to *P. suberosa* plants (500.33 μg RE per plant) under LI-50 treatment.

Significant decreases in OR concentration were noted in Tainung No. 1 plants as the light intensity decreased, but in *P. suberosa* plants, a significant decrease in OR concentration was observed with LI-50 treatment (151.46 μg (g DW)^−1^) compared to other light treatments (184.81–187.50 μg (g DW)^−1^). Furthermore, *P. suberosa* plants had significantly higher OR contents than Tainung No. 1 plants in response to both LI-50 and LI-15 treatments. When the OR content was calculated on the plant level, a higher OR content was detected in Tainung No. 1 (25.53 μg per plant) and *P. suberosa* (31.73 μg per plant) under LI-100 treatment compared to LI-15 treatment. Moreover, under LI-15 treatment, significantly lower OR contents per plant were detected in the Tainung No. 1 species (5.69 μg per plant) compared to the *P. suberosa* species (22.29 μg per plant).

IV concentration of both species under LI-100 and LI-50 treatments were significantly higher than those under LI-15 treatment. In particular, *P. suberosa* plants under LI-100 treatment (687.18 μg (g DW)^−1^) displayed a significant 20-fold increase compared to LI-15 treatment (34.05 μg (g DW)^−1^), and it was also significantly higher (an 11-fold increase) than that of Tainung No. 1 plants (62.05 μg (g DW)^−1^). When the IV content was calculated on the plant level, the patterns and trends of IV contents per plant of *P. suberosa* appeared similar to those of OR contents under various light treatments. Notably, the *P. suberosa* species under LI-100 treatment (103.92 μg per plant) exhibited a 31-fold increase compared to LI-15 treatment (3.26 μg per plant). When species were compared across light treatments, Tainung No. 1 plants (10.14 μg per plant) under LI-15 treatment exhibited significantly higher IV contents than did *P. suberosa* plants (3.26 μg per plant).

Leaves of *Passiflora* species are very popular vegetables in Taiwan and are treated as herbaceous plants, functional foods, and nutraceutical products. In our study, the hot-water PLE was analyzed by C18-HPLC; thus, utilization of PLE offers the possibility of being a natural food additive and food preservative and could be developed as a functional food and for nutraceutical use. Myriad environmental factors influence plant growth and directly impact biosynthetic pathways, thus affecting the secondary metabolism of bioactive compounds, and environmental conditions during plant growth may affect certain biosynthetic pathways that lead to variabilities in individual secondary metabolites [43]. Many of them play key roles in plants’ adaptation to abiotic stresses, and it is very important to keep a balance between biomass yields and metabolic compound concentrations to maximize economic benefits [44]. Therefore, quantifying the optimal stress level of a single environmental factor is crucial for the actual production of medicinal plants in a controlled environment. Hikosaka et al. [45] reported that greenhouse cultivation is an effective method for the steady production of medicinal plants because lighting conditions can be suitably controlled for plant growth and quality, and it is possible that concentrations of bioactive compounds in medicinal plants can be increased through environmental control in a greenhouse.

Photosynthesis is sensitive to environmental changes, and under natural conditions, photosynthesis is biochemically regulated in response to environmental changes to maintain a balance between the rates of component processes and secondary metabolite concentrations [46]. Increases in all tested metabolic contents of Tainung No. 1 were clear under LI-50 treatment compared to LI-15 treatment, and these can be used as a health food and for medicinal purposes due to high secondary metabolite contents. In *P. suberosa* plants, LI-15 treatment produced a higher TP content than other light treatments, but higher TF, OR, and IV contents were detected in LI-100 treatment compared to other light treatments. These differences can possibly be attributed to evolutionary adaptations of various species to growth under different light intensities. *Passiflora suberosa* is native to forests, and so, high light irradiances may be adverse for this species and more secondary metabolites may be produced in full light. Wide variations in secondary metabolite contents were observed in the two varieties, with *P. suberosa* possessing higher TP and TF contents than Tainung No. 1 in each lighting treatment, except for the TF content (per plant) with LI-50 treatment. In general, OR and IV contents (per leaf DW) of *P. suberosa* under LI-50 treatment were higher than those in Tainung No. 1. LI-100 treatment would be beneficial for Tainung No. 1 plants to form flower buds and blossoms and to inhibit leaf growth and development, whereas LI-50 treatment would not favor reproductive growth but would be beneficial for vegetative growth and synthesis of secondary metabolites from excess photosynthetic nutrients. Consequently, LI-100 and LI-50 would respectively be beneficial for fruit production and functional ingredients. Light intensity influences the growth and photosynthetic potential of *Passiflora* plants, and different responses by metabolite compounds in leaves depend on the variety of *Passiflora*, which can be used to optimize the growth and development of plants in controlled light settings. Levels of secondary metabolites were influenced by the availability of light, and fluctuations in the contents of metabolites are a plant’s response to environmental factors and part of an adaptive strategy leading to tolerance to light irradiance. However, the conditions of the natural habitat, biosynthesis, and accumulation of TP, TF, OR, and IV by *Passiflora* leaves in response to light irradiance are not yet completely clear. Future studies should attempt to explain the mechanisms of changes in the contents of TP, TF, OR, and IV of *Passiflora* species grown in different light environments. These results also signify the complex relationships between light intensity and metabolite biosynthesis, and further studies are necessary to better understand factors regulating the synthetic balance of the four metabolites in the flavonoid pathway.

Light intensity affects the accumulation of phenolic and flavonoid contents of a plant. This is possible because every plant has a specific light requirement for the maximum production of flavonoid and phenolic compounds. Different *Passiflora* plants subjected to various light intensities can be used as health foods and for medicinal purposes due to their high secondary metabolite contents; therefore, they could be used to develop physiological markers to select germplasm through genetic manipulation. In the cases of the *P. suberosa* variety with the highest concentration of TP (3921 μg RE per plant) under LI-15 treatment, of TF (4611.73 μg RE (g DW)^−1^) under LI-100 treatment, and of IV (687.18 μg (g DW)^−1^) under LI-100 treatment and the Tainung No. 1 variety with the highest content of OR (199.48 μg (g DW)^−1^) under LI-100, they share an initial common biosynthetic pathway that is normally present and is suggested for further breeding/cultivation and bioactivity study. TP, TF, OR, and IV contents may be the rate-limiting factors of metabolites for use as genetic tools to develop an effective method for selecting *Passiflora* varieties to improve adaptability to light irradiance, and a better understanding of the relationships of leaf growth traits and physiological parameters with TP, TF, OR, and IV contents will stimulate more efficient breeding of *Passiflora* plants. These may be useful in screening for shading-tolerant plants, and different lighting culture systems may achieve commercial *Passiflora* plant production by utilizing rapid, large-scale, precise management practices. The selection of cultivars for specific light-intensity practices is a preharvest factor that can significantly influence secondary metabolite contents of *Passiflora* plants.

## 3. Materials and Methods

### 3.1. Plant Materials and Cultural Practice

Passion fruit (*P. edulis* × *P. edulis* f. *flavicarpa*) “Tainung No. 1”, the main variety cultivated in Taiwan, is a commercial hybrid and is widely used by the juice industry. The “Tainung No. 1” passion fruit is a crossbreed in which a scion of the yellow passion fruit was grafted onto the rootstock of the purple passion fruit in 1974 at Taiwan Fengshan Institute of Tropical Horticulture. This plant tolerates a wide range of climatic conditions from sea level to altitudes of up to 600 meters, is cultivated on a large scale, and is the most popular passion fruit in Taiwan [47]. *Passiflora suberosa* L. is a Taiwanese vegetable cultivated by family farming as an important medicinal plant to treat several diseases, and leaves from this plant are consumed as a fresh green vegetable. This species was identified, and a voucher specimen was deposited with code 204998, *Flora of Taiwan* [48]. Healthy plants of these two varieties were obtained from local shops in Taipei, Taiwan and grown in an open field at National Taiwan University (NTU, latitude 25.01°N) in February 2019. When plants were 15–17 cm tall with four to five leaves, they were transplanted into 20-cm plastic pots (2 L) containing commercial potting soil with a substrate mixture of vermiculite:peat:soil (3:3:4, v/v/v) and were placed in an environmentally controlled greenhouse at NTU under an 8-h photoperiod at 28/22 °C day/night temperatures and a relative humidity of 80%. They were evenly spaced to encourage similar growth rates and sizes. Plants were watered twice a week, and an optimal amount of a compound water-soluble fertilizer solution (Scott, Marysville, OH, USA), of 20N-8.8P-16.6K at 1 g L^−1^, was applied weekly. Plants were grown for 60 days, and those with a uniform size were selected and randomly separated into different groups for the light-intensity experiments.

### 3.2. Light-Intensity Measurements

Plants were divided into three groups for each light-intensity treatment, and ten replicates of each treatment were arranged in a completely randomized design; 30 pots in total of each variety were used. Three light-intensity levels were created by blocking light penetration using black shading nets stretched over a rigid frame. The light-intensity groups were 100% light intensity (non-shaded, as the control, LI-100), 50% light intensity (50% shaded, LI-50), and 15% light intensity (85% shaded, LI-15). Fluorescent lights were used as a light source. The incidence of photosynthetically active radiation in microeinsteins per square meter per second (µE m^−2^ s^−1^) was measured with the LI-250 portable Light Meter System equipped with a Linear Quantum Sensor LI-191SA (LI-COR Bioscience, Lincoln, NE, USA). The average light intensities in the three treatments were 1396, 619, and 187 μmol m^−^^2^ s^−^^1^ of photosynthetic photon flux density (PPFD) at noontime (April to June 2019) for the LI-100, LI-50, and LI-15 treatments, respectively. Microclimate stations were centrally located within each study plot where values of PPFD were recorded. The culture conditions were the same for each group as those mentioned above for the environmentally controlled greenhouse, where the average temperature was 25.4 °C and photoperiod was 12.5 h, during the 2-month period of the light-intensity experiments.

### 3.3. Plant Growth Measurements

Observations of the number of leaves per plant and length, width, and area of the leaves were recorded 60 days after the experiment was established. Leaf area (LA) was measured with a portable LAI-3000C instrument (LI-COR). The dry weight (DW) of leaves was measured with an electronic balance. For each treatment, healthy and fully expanded leaves were taken and dried in a drying oven for 48 h at 70 °C before weighing.

### 3.4. Measurement of Photosynthetic Parameters

Healthy, fully expanded mature leaves from the middle portion of each plant were used to determine total chlorophyll (Chl) contents using a soil-plant analysis development (SPAD) analyzer (SPAD-502 Chlorophyll Meter, Konica Minolta, Tokyo, Japan).

The potted plants were moved to the shade under a cottage before sunrise at 05:30–06:00, and then, the ChlF parameters of dark-adapted leaves were measured with a portable fluorometer (MINI-PAM, Walz, Effeltrich, Germany) at ambient temperature after adaptation to the dark for 20 min. The middle portions of fully expanded third leaves of each plant were used for the measurements. Values of the minimal ChlF (Fo) and maximal ChlF (Fm) of dark-adapted samples were respectively determined using modulated irradiation of a weak LED beam (measuring light) and a saturating pulse. We then calculated the maximum photochemical quantum yield (Fv/Fm), where Fv, the yield of variable fluorescence, was calculated as (Fm − Fo). When measuring Fv/Fm, samples were first acclimated to dark conditions to ensure that all reaction centers were in an open state, and there was minimal non-photochemical dissipation of excitation energy [49]. Measurements were recorded using WinControl-3 software (Heinz Walz, Effeltrich, Germany).

The photosynthetic rate measurement was estimated with a portable, open-flow gas exchange system (LI-6400XT, LI-COR) connected to a leaf chamber and LED light source at 25 °C, a CO_2_ concentration of 400 μmol·mol^−1^, and a relative humidity of 55%. PPFD was set to 1000 μmol·m^−2^·s^−1^ radiation. The leaf gas exchange measurements were performed in the middle portion of fully expanded third leaves of each plant, and all measurements were taken before 14:00 to avoid the midday depression in photosynthesis. CO_2_ exchange values were recorded every 2 min until stable (about 10 min under each level of illumination). Values of the net photosynthetic rate (Pn), transpiration rate (E), stomatal conductance to water vapor (Gs), intercellular CO_2_ concentration (Ci), and atmospheric CO_2_ concentration (Ca) were simultaneously recorded and calculated using values of CO_2_ and humidity (55%) inside the chamber. The operation was automatic, and data were stored in the computer within the console and analyzed by “Photosyn Assistant” software.

### 3.5. Determination of TP and TF Contents and Flavone Concentrations

After 60 days of shading treatments, the middle portions of the fully expanded third leaves of two *Passiflora* species were collected and first rinsed with running water and then with distilled water. The washed leaves were next placed in clean envelopes and dried for 30 min at 105 °C in an electric blast drying oven. They were dried at 80 °C to a constant weight, and the dried leaves were ground up with a grinder. About 1 g of a dry powdered leaf sample was immersed in 10 mL of hot water (90 °C) in a water bath at 90 °C for 15 min. The liquid phase was then separated from debris using a syringe filter (13 mm × 0.22 μm, Millex-GV, Millipore, Sigma Aldrich, St. Louis, MO, USA) under a vacuum (Rapidvp Vacuum Evaporation System, Labconco, Kansas City, MO, USA) to obtain crude *Passiflora* leaf extract (PLE), which was used for analysis of TP, TF, and flavone concentrations.

The TP content was determined as previously described [50]. Standard gallic acid and an aliquot of PLE were diluted with an acidified methanol solution containing 1% HCl. Two milliliters of 2% Na_2_CO_3_ was mixed into each 100-μl sample and allowed to equilibrate for 2 min before adding 50% Folin-Ciocalteau reagent (Sigma Aldrich). Absorbance at 730 nm was measured at room temperature using a microplate reader (InterMed, South Portland, ME, USA). A standard curve for gallic acid was used to calculate TP levels. The TP content was expressed as mg gallic acid equivalent (GAE) (g of dry weight (DW))^−1^. The standard curve equation was y = 0.4995x − 0.011, where *R*^2^ = 0.9950. The assay was run in triplicate for each sample.

TFs in extracts were determined by the method of Saravanan and Parimelazhagan [51]. Briefly, 1 mL of PLE (5 mg·mL^−1^) was mixed with 1 mL aluminum chloride (5%). The mixture was stirred and kept at room temperature for 15 min. The absorbance was measured at 500 nm on a spectrophotometer. Rutin was used as a reference standard, and the TF content was expressed as milligrams of rutin equivalent (RE) per gram of DW (μg RE·g^−1^ DW). The linear range of the calibration curve was 6.25–200 μg mL^−1^ (*R*^2^ = 0.9942).

To separate and identify flavone compounds in the PLE, reverse-phase C18 high-performance liquid chromatography (HPLC) was used as described by Pereira et al. [52]. A Hypersil ODS C18 column (250 × 4.6 mm, 5 μm) was connected to the LC-200 HPLC system (Perkin-Elmer, Waltham, MA, USA) and equilibrated with 0.05% aqueous trifluoroacetic acid. Ten microliters of the hot water-extracted PLE was used for the HPLC analysis after filtration through a 0.22-μm syringe filter (Millex-GV, Millipore, Sigma Aldrich), which was injected and eluted with 0.1% aqueous formic acid and acetonitrile. The flow rate was 1 mL min^−1^ at 30 °C. Collected fractions of the eluent were all in 1-mL aliquots, and the eluent’s absorbance at 345 nm was scanned with a LC-785A UV/VIS detector (Perkin-Elmer). Peaks were identified by comparing the retention time and ultraviolet absorption spectrum of the eluting peaks with flavone standards. Authentic standards for orientin (OR) and isovitexin (IV) were used to identify the flavone compounds of *Passiflora* species. A series of standard solutions ranging 0.625–100 µg·mL^−1^ was tested to determine the calibration curve. Regression equations for OR and IV were calculated in the form of y = ax + b, where y and x were the peak area and amount of standard injected, respectively, and all calibration curves had coefficients of linear correlation *r*^2^ of >0.990.

### 3.6. Statistical Analysis

All measurements were evaluated for significance using an analysis of variance (ANOVA) followed by a least significant difference (LSD) test and *t*-test at *p* ≤ 0.05. All statistical analyses were conducted using CoStat 6.4 (CoHort Software, Monterey, CA, USA).

## 4. Conclusions

Two different *Passiflora* cultivars displayed variations in leaf growth traits, ChlF, SPAD, photosynthesis capacity parameters, and secondary metabolite contents, and differences in each cultivar were associated with lighting responses. Tainung No. 1 plants, a shade-tolerant species, under LI-15 condition exhibited markedly higher leaf growth traits, Fo, Fm, SPAD, and Ci/Ca values and lower Pn and E values compared to the LI-100 condition. However, *P. suberosa* plants suffered from LI-15 treatment with significantly lower leaf numbers and DW compared to the LI-100 condition. Furthermore, the SPAD, Fo, and Fm developed in this study for evaluating and screening leaf growth, photosynthesis, and transpiration parameters of *Passiflora* plants under shading treatments are a nondestructive and readily applicable way to assess early growth on a large scale in open fields. The light intensity can be an efficient way to maintain the sustainability of light resources and to enrich TP, TF, OR, and IV contents, and the cultivation of *Passiflora* plants for use as crude medicinal materials is proposed. Increases in all tested secondary metabolites of Tainung No. 1 were clear with LI-50 treatment compared to LI-15 treatment, whereas high light irradiance in LI-100 may be adverse for *P. suberosa* and may cause more secondary metabolites to be produced in full light due to its natural habitat being in the forest. Differential adaptations of the two studied varieties concerning individual morphophysiological plasticity may be essential tools for plants to adapt to heterogeneous light environments. This information will be very useful for *Passiflora* producers when selecting varieties and when applying strategies of light intensity to achieve their goals in terms of leaf production, physiological parameters, and the accumulation of secondary metabolite contents, and we suggest LI-50 of 619 μmol m^−^^2^s^−^^1^ PPFD for Tainung No. 1 commercial production in indoor farming to minimize energy costs while maintaining high leaf yields and secondary metabolite contents.

## Figures and Tables

**Table 1:** ANOVA results of the main effects of variety (V), light intensity (L), and their interaction effect (L × V) on the leaf growth and physiological parameters of of Tainung No. 1 and *Passiflora suberosa* plants.

Source of Variation	Significance												
	Leaf Number	Leaf Length (cm)	Leaf Width (cm)	Leaf Area (cm^2^)	Dry Weight (g/Leaf)	Fo	Fm	Fv/Fm	SPAD Value	Pn	E	Gs	Ci/Ca
Variety (V)	***	***	**	***	NS	NS	*	NS	*	NS	NS	NS	NS
Light intensity (L)	**	***	**	***	***	*	*	NS	***	*	**	*	*
L × V	***	***	NS	**	NS	*	NS	NS	*	NS	NS	NS	NS

NS, nonsignificant; * *p* ≤ 0.05, ** *p* ≤ 0.01 and *** *p* ≤ 0.001; Fo, minimal fluorescence; Fm, maximal fluorescence; Fv/Fm, maximum photochemical efficiency of photosystem II; SPAD, soil-plant analysis development; Pn, net photosynthetic rate; E, the transpiration rate; Gs, stomatal conductance; Ci/Ca, intercellular-to-atmospheric CO_2_ concentration ratio.

**Table 2:** ANOVA results of the main effects of variety (V), light intensity (L), and their interaction effect (L × V) on the secondary metabolites of Tainung No. 1 and *Passiflora suberosa* plants.

Source of Variation	Significance							
	Total Phenol Concentration (μg GAE (g DW)^−1^)	Total Phenol Content (μg GAE Per Plant)	Total Flavonoid Concentration (μg RE (g DW)^−1^)	Total Flavonoid Content (μg RE Per Plant)	Orientin Concentration (μg (g DW)^−1^)	Orientin Content (μg Per Plant)	Isovitexin Concentration (μg (g DW)^−1^)	Isovitexin Content (μg Per Plant)
Variety (V)	NS	***	***	***	***	***	***	***
Light intensity (L)	***	**	***	***	***	***	***	***
L × V	*	**	*	***	***	***	***	***

NS, nonsignificant; * *p* ≤ 0.05, ** *p* ≤ 0.01 and *** *p* ≤ 0.001.

## Supplementary Materials

The following are available online at https://www.mdpi.com/2223-7747/9/5/633/s1.
