# Supplementary material for: Flavonoid Compounds and Photosynthesis in Passiflora Plant Leaves under Varying Light Intensities
Source: Plants (Basel). 2020 May 15;9(5):633. doi: 10.3390/plants9050633 (PMC7285318; doi:10.3390/plants9050633)
Supplement: Supplementary file 1 [file plants-09-00633-s001.pdf]

Table S1. Effects of light intensity on the leaf number, length, width, and dry weight of Tainung No. 1 and *Passiflora suberosa* plants.

Variety	Light intensity treatment	Leaf number	Leaf length (cm)	Leaf width (cm)	Leaf area (cm^2)	Dry weight (g/leaf)
Tainung No. 1	LI-100	10.67 ± 1.51 bB	7.94 ± 1.06 bA	7.58 ± 1.55 bA	32.28 ± 9.44 bA	0.11 ± 0.04 bA
	LI-50	16.33 ± 1.51 aB	9.58 ± 1.75 bA	9.68 ± 2.13 abA	49.88 ± 16.68 bA	0.17 ± 0.08 aA
	LI-15	16.50 ± 1.05 aB	12.61 ± 1.25 aA	10.63 ± 1.79 aA	71.63 ± 16.24 aA	0.17 ± 0.06 aA
P. suberosa	LI-100	23.17 ± 1.94 aA	5.95 ± 0.37 bB	7.57 ± 0.52 bA	21.21 ± 2.56 bB	0.08 ± 0.01 aA
	LI-50	21.50 ± 1.38 aA	6.67 ± 0.36 aB	8.07 ± 0.31 abA	25.35 ± 2.26 aB	0.07 ± 0.01 abB
	LI-15	18.50 ± 0.84 bA	6.95 ± 0.22 aB	8.42 ± 0.45 aB	27.50 ± 1.86 aB	0.06 ± 0.02 bB
Source of variation						
Variety (V)		***	***	**	***	NS
Light intensity (L)		**	***	**	***	***
L × V		***	***	NS	**	NS

Data were recorded and calculated after 2 months of light-intensity treatment of six replicates. Means in the same column within a light treatment followed by different lowercase letters significantly differ at $p \leq 0.05$ by the least significant difference (LSD) test. Means in the same column within the same light treatment of the two species followed by different capital letters significantly differ at $p \leq 0.05$ by the LSD test. Each treatment was assumed to be dependent on the other. ANOVA results of the main effects of variety (V), light intensity (L), and their interaction effect (L × V) on the leaf growth of Passiflora species are shown as NS, nonsignificant; ** $p \leq 0.01$ and *** $p \leq 0.001$. LI-100 (100% light intensity, non-shaded, as the control), LI-50 (50% light intensity, 50% shaded), and LI-15 (15% light intensity, 85% shaded) represent for the average light intensity passing through in each treatment at 1396, 619, and 187 µmol m−2s−1 PPFD, respectively.

Table S2. Effects of light intensity on minimal fluorescence (Fo), maximal fluorescence (Fm), maximum photochemical efficiency of photosystem II (Fv/Fm), and soil-plant analysis development (SPAD) values of Tainung No. 1 and *Passiflora suberosa* plants.

Variety	Light intensity treatment	Fo	Fm	Fv/Fm	SPAD value
Tainung No. 1	LI-100	345.83 ± 18.98 bB	1674.17 ± 185.13 bB	0.78 ± 0.01 aA	29.00 ± 3.03 aB
	LI-50	364.07 ± 21.09 bA	1687.67 ± 93.11 bB	0.79 ± 0.01 aA	32.35 ± 1.47 aB
	LI-15	396.67 ± 32.13 aA	1989.33 ± 216.22 aA	0.80 ± 0.03 aA	34.27 ± 1.96 aA
P. suberosa	LI-100	381.67 ± 18.40 aA	1863.83 ± 133.91 aA	0.79 ± 0.02 aA	36.66 ± 2.92 aA
	LI-50	383.17 ± 15.38 aA	1942.33 ± 117.48 aA	0.80 ± 0.01 aA	37.10 ± 1.66 aA
	LI-15	388.17 ± 24.60 aA	1985.50 ± 256.34 aA	0.81 ± 0.02 aA	37.26 ± 1.12 aA
Source of variation					
Variety (V)		NS	*	NS	*
Light intensity (L)		*	*	NS	***
L × V		*	NS	NS	*

Data were recorded and calculated after 2 months of light-intensity treatment of six replicates. Means in the same column within a light treatment followed by different lowercase letters significantly differ at $p \leq 0.05$ by the least significant difference (LSD) test. Means in the same column within the same light treatment of the two species followed by different capital letters significantly differ at $p \leq 0.05$ by the LSD test. Each treatment was assumed to be dependent on the other. ANOVA results of the main effects of variety (V), light intensity (L), and their interaction effect (L × V) on the leaf growth of Passiflora species are shown as NS, nonsignificant; * $p \leq 0.05$ and *** $p \leq 0.001$. LI-100 (100% light intensity, non-shaded, as the control), LI-50 (50% light intensity, 50% shaded), and LI-15 (15% light intensity, 85% shaded) represent for the average light intensity passing through in each treatment at 1396, 619, and 187 μmol m−2s−1 PPFD, respectively.

Table S3. Effects of light intensity on the net photosynthetic rate (Pn), transpiration rate (E), stomatal conductance to water vapor (Gs), and intercellular to atmospheric CO_2 concentration ratio (Ci/Ca) of Tainung No. 1 and *Passiflora suberosa* plants.

**Variety**	**Light intensity treatment**	**Pn (μmol·CO_2·m^{-2}·s^{-1})**	**E (mmol·H_2O·m^{-2}·s^{-1})**	**Gs (mol·m^{-2}·s^{-1})**	**Ci/Ca**
Tainung No. 1	LI-100	1.28 ± 1.51 aA	0.43 ± 0.05 aA	0.02 ± 0.02 aA	0.65 ± 0.19 bA
	LI-50	1.84 ± 1.13 aA	0.50 ± 0.03 aA	0.02 ± 0.01 aA	0.71 ± 0.12 abA
	LI-15	0.68 ± 0.45 aB	0.27 ± 0.07 bB	0.01 ± 0.01 aB	0.77 ± 0.10 aA
P. suberosa	LI-100	1.16 ± 0.41 aA	0.52 ± 0.06 bA	0.02 ± 0.01 aA	0.63 ± 0.23 aA
	LI-50	1.52 ± 0.65 aA	0.60 ± 0.11 abA	0.02 ± 0.01 aA	0.64 ± 0.07 aA
	LI-15	2.14 ± 0.67 aA	0.76 ± 0.16 aA	0.03 ± 0.01 aA	0.62 ± 0.05 aB
Source of variation					
Variety (V)		NS	NS	NS	NS
Light intensity (L)		*	**	*	*
L × V		NS	NS	NS	NS

Data were recorded and calculated after 2 months of light-intensity treatment of six replicates. Means in the same column within a light treatment followed by different lowercase letters significantly differ at $p \leq 0.05$ by the least significant difference (LSD) test. Means in the same column within the same light treatment of the two species followed by different capital letters significantly differ at $p \leq 0.05$ by the LSD test. Each treatment was assumed to be dependent on the other. ANOVA results of the main effects of variety (V), light intensity (L), and their interaction effect (L × V) on the leaf growth of Passiflora species are shown as NS, nonsignificant; * $p \leq 0.05$ and ** $p \leq 0.01$. LI-100 (100% light intensity, non-shaded, as the control), LI-50 (50% light intensity, 50% shaded), and LI-15 (15% light intensity, 85% shaded) represent for the average light intensity passing through in each treatment at 1396, 619, and 187 μmol m−2s−1 PPFD, respectively.

Table S4. Effects of the light intensity on contents of total phenol, total flavonoid, orientin, and isovitexin per leaf and per plant in Tainung No. 1. and *Passiflora suberosa* plants.

Variety	Light intensity treatment	Total phenol concentration (μg GAE (g DW)$^{-1}$)	Total phenol content (μg GAE per plant)	Total flavonoid concentration (μg RE (g DW)$^{-1}$)	Total flavonoids content (μg RE per plant)	Orientin concentration (μg (g DW)$^{-1}$)	Orientin content (μg per plant)	Isovitexin Concentration (μg (g DW)$^{-1}$)	Isovitexin content (μg per plant)
Tainung No. 1	LI-100	1001.89 ± 28.41 aB	187.90 ± 30.49 cB	3719.78 ± 170.74 aB	664.98 ± 70.99 bA	199.48 ± 50.08 aA	25.53 ± 5.20 aA	62.05 ± 3.85 aB	11.58 ± 2.11 bB
	LI-50	1048.48 ± 25.85 aA	364.85 ± 20.52 aB	2666.89 ± 402.16 bB	934.07 ± 56.60 aA	89.39 ± 11.98 bB	23.90 ± 2.04 aA	53.79 ± 5.14 bB	15.80 ± 3.16 aA
	LI-15	1014.90 ± 64.56 aB	294.76 ± 39.62 bB	1072.97 ± 136.06 cB	277.68 ± 42.02 cA	25.71 ± 3.34 cB	5.69 ± 0.72 bB	37.28 ± 0.62 cA	10.14 ± 1.09 bA
P. suberosa	LI-100	1073.27 ± 37.47 bA	2786.73 ± 224.65 bA	4611.73 ± 480.63 aA	673.06 ± 20.16 aA	187.50 ± 17.62 aA	31.73 ± 2.73 aA	687.18± 144.28 aA	103.92±19.11 aA
	LI-50	1121.33 ± 76.33 abA	2717.29 ± 186.18 bA	3762.73 ± 305.40 bA	500.33 ± 41.60 bB	151.46 ± 14.86 bA	22.61 ± 0.94 bA	139.80 ± 23.65 bA	19.52 ± 2.76 bA
	LI-15	1196.62 ± 99.13 aA	3921.66 ± 808.59 aA	2733.10 ± 149.55 cA	291.68 ± 51.24 cA	184.81 ± 6.46 aA	22.29 ± 4.77 bA	34.05 ± 3.87 cA	3.26 ± 0.23 cB
Source of variation									
Variety (V)		NS	***	***	***	***	***	***	***
Light intensity (L)		***	**	***	***	***	***	***	***
L × V		*	**	*	***	***	***	***	***

Data were recorded and calculated after 2 months of light-intensity treatment of six replicates. Means in the same column within a light treatment followed by different lowercase letters significantly differ at $p \leq 0.05$ by the least significant difference (LSD) test. Means in the same column within the same light treatment of the two species followed by different capital letters significantly differ at $p \leq 0.05$ by the LSD test. Each treatment was assumed to be dependent on the other. ANOVA results of the main effects of variety (V), light intensity (L), and their interaction effect (L × V) on the leaf growth of Passiflora species are shown as NS, nonsignificant; * $p \leq 0.05$, ** $p \leq 0.01$, and *** $p \leq 0.001$. LI-100 (100% light intensity, non-shaded, as the control), LI-50 (50% light intensity, 50% shaded), and LI-15 (15% light intensity, 85% shaded) represent for the average light intensity passing through in each treatment at 1396, 619, and 187 μmol m−2s−1 PPFD, respectively.
